# The Importance of the Dissociation Rate in Ion Channel Blocking

**DOI:** 10.3389/fncel.2018.00033

**Published:** 2018-02-09

**Authors:** Hugo Zeberg, Johanna Nilsson, Peter Århem

**Affiliations:** ^1^Department of Neuroscience, Karolinska Institutet, Stockholm, Sweden; ^2^Department of Clinical Neuroscience, Karolinska Institutet, Stockholm, Sweden

**Keywords:** ion channel block, voltage-clamp, dissociation rate constant, peak current, Markov chain model, Monte Carlo simulation

## Abstract

Understanding the relationships between the rates and dynamics of current wave forms under voltage clamp conditions is essential for understanding phenomena such as state-dependence and use-dependence, which are fundamental for the action of drugs used as anti-epileptics, anti-arrhythmics, and anesthetics. In the present study, we mathematically analyze models of blocking mechanisms. In previous experimental studies of potassium channels we have shown that the effect of local anesthetics can be explained by binding to channels in the open state. We therefore here examine models that describe the effect of a blocking drug that binds to a non-inactivating channel in its open state. Such binding induces an inactivation-like current decay at higher potential steps. The amplitude of the induced peak depends on voltage and concentration of blocking drug. In the present study, using analytical methods, we (i) derive a criterion for the existence of a peak in the open probability time evolution for a model with an arbitrary number of closed states, (ii) derive formula for the relative height of the peak amplitude, and (iii) determine the voltage dependence of the relative peak height. Two findings are apparent: (1) the dissociation (unbinding) rate constant is important for the existence of a peak in the current waveform, while the association (binding) rate constant is not, and (2) for a peak to exist it suffices that the dissociation rate must be smaller than the absolute value of all eigenvalues to the kinetic matrix describing the model.

## Introduction

Understanding the relationships between the rates and dynamics of current wave forms under voltage clamp conditions is essential for understanding phenomena such as state-dependence and use-dependence, which are fundamental for the action of drugs used as anti-epileptics, anti-arrhythmics, and anesthetics. In the present study, we mathematically analyze models of open state blocking mechanisms previously suggested for the local anesthetic bupivacaine action on Kv channels (Longobardo et al., 2001; Nilsson et al., 2003, 2008).

The dynamics of ion channels are generally considered to be memory-less (i.e., they possess the Markov property) and can be analyzed in terms of Markov chains (Colquhoun and Hawkes, 1995). We, therefore, explore Markov-chain type kinetic schemes, describing open state dependent drug-binding. Both analytical and Monte Carlo methods are used. We derive criteria for the existence of an induced current peak and formula for the peak height and its dependence on membrane potential. It should be noted that similar serial Markov chains can be used to describe a number of other pharmacological processes, such as competitive binding of antagonists or agonists, and, consequently, insights from the present analysis can also be of value in these cases.

Two findings are apparent from this study: (i) the dissociation (unbinding) rate constant is important for the existence of a peak in the current waveform, while the association (binding) rate constant is not, and (ii) for a peak to exist it suffices that the dissociation rate must be smaller than the absolute value of all eigenvalues to the kinetic matrix describing the model.

That the criterion is independent of the association rate (as long as it is greater than zero) has the implication that the concentration of the local anesthetic does not influence the existence of a peaked waveform under voltage clamp.

## Methods

All ion channels, either voltage-gated or ligand-gated, open and close randomly, and an accurate explanation of their behavior must, therefore, be of a probabilistic character (Colquhoun and Hawkes, 1995; Johnston and Wu, 1995). In this work, we use analytical and Monte Carlo methods to analyze Markov chain models of ion channels, describing the probability of the channel being in each state. We investigate the following three-state Markov scheme using analytical methods

(1) $$\begin{array}{r} C\;\begin{array}{c} \alpha \\ ⇄\;\\ \beta \end{array}O\;\begin{array}{c} \gamma \\ ⇄\;\\ \delta \end{array}B \end{array}$$

where *C, O*, and *B* denote closed, open and blocked states, respectively, where α, β, γ, and δ denote rate constants, and where γ includes the concentration of the blocking drug. We used the terms κ x *L* and λ for γ and δ, respectively. We also analyze extended versions of Scheme 1, namely

(2) $$\begin{array}{r} C\begin{array}{c} m\alpha \\ ⇄\;\\ \beta \end{array}\ldots ⇄\;C\begin{array}{c} \alpha \\ ⇄\;\\ m\beta \end{array}O\begin{array}{c} \gamma \\ ⇄\;\\ \delta \end{array}B \end{array}$$

This extended kinetic scheme describes a channel system with *m* equal and independent gates in accordance with the Hodgkin-Huxley formalism for Kv channels (as first noted by Richard Fitzhugh, 1961). With this notation, the number of states in the scheme is *n* = *m* + 2.

## Results

### The existence of a current peak during voltage clamp: analysing a three-state scheme

Time-dependent effects of bupivacaine on different Kv channels at + 60 mV at different concentrations have been analyzed in several studies, using voltage clamp technique (see Gonzalez et al., 2001; Nilsson et al., 2003). As can be inferred from these studies, a current peak can be observed at higher voltages. The voltage dependence of this induced peak, however, has been surprisingly little studied (but see Gonzalez et al., 2001).

We seek to find sufficient and necessary prerequisites for the existence of such a peak. We will first derive this for a three-state scheme, then use similar techniques to derive criteria for the four-state scheme, and in the Appendix for an n-state scheme. The techniques are generally those of dynamical systems and matrix algebra, which we (Gouwens et al., 2010; Zeberg et al., 2010, 2015; Sahlholm et al., 2016) and others (for an overview see Koch, 2004; Izhikevich, 2007) have used extensively for analyzing these systems. The differential equation for Scheme 1 written in matrix form is **x**′ = **Ax** where

(3) $$\begin{array}{c} \text{x}(t)=\left[\begin{array}{c} C(t)\\ O(t)\\ B(t) \end{array}\right]\;\text{A}=\left[\begin{array}{ccc} -\alpha & \beta & 0\\ \alpha & -(\beta +\gamma ) & \delta \\ 0 & \gamma & -\delta \end{array}\right] \end{array}$$

with the general solution

(4) $$\begin{array}{r} \text{x}\left(t\right)=c_{1}{\text{V}}_{1}e^{r_{1}t}+c_{2}{\text{V}}_{2}e^{r_{1}t}+c_{3}{\text{V}}_{3}e^{r_{3}t} \end{array}$$

and where **V**_*i*_ and *r*_1_ are the eigenvectors and eigenvalues of the transition matrix **A**. *c*_*i*_ are constants dependent on the initial conditions. Experimentally, voltage clamp experiments are typically done with a strongly negative potential as an initial condition, meaning that all ion channels are in the (first) closed state, i.e.,

(5) $$\text{x}(0)=\left[\begin{array}{c} 1\\ 0\\ 0 \end{array}\right]$$

We will now use the Putzer algorithm to solve *O*(*t*). The Putzer Algorithm is a method for analytically evaluating matrix exponentials using only eigenvalues and components in the solution of a relatively simple linear system (Putzer, 1966). This approach might seem a little bit cumbersome for solving the three-state scheme but will later enable us to solve not just the four-state model but also the general case (complete proof given in the Appendix). By the Putzer algorithm, the solution to **x**′(*t*) = **Ax**(**t**), where **A** is a 3 × 3 matrix, can be written on the form

(6) $$\begin{array}{r} \text{x}\left(t\right)=\left(p_{1}\left(t\right){\text{M}}_{1}+p_{2}\left(t\right){\text{M}}_{2}+p_{3}\left(t\right){\text{M}}_{3}\right)\text{x}\left(0\right) \end{array}$$

Where *p*_*i*_(*t*) and **M**_1_ are defined as follows. Define 3 × 3 matrices **M**_1_, **M**_2_, and **M**_3_ by the formula

(7) $$\begin{array}{r} {\text{M}}_{1}=I\;{\text{M}}_{i}=\left(\text{A}-r_{i-1}I\right){\text{M}}_{i-1}\;i=2,3 \end{array}$$

and let the functions *p*_1_(*t*), *p*_2_(*t*), and *p*_3_(*t*) be given by solutions to the differential system

(8) $$\begin{array}{rcl} {p_{1}}^{\prime }\left(t\right) & = & r_{i}p_{1}\left(t\right)\;{\;p}_{1}\left(0\right)=1\\ {p_{2}}^{\prime }\left(t\right) & = & r_{2}p_{2}\left(t\right)+p_{1}\left(t\right)\;p_{2}\left(0\right)=0\\ {p_{3}}^{\prime }\left(t\right) & = & r_{3}p_{3}\left(t\right)+p_{2}\;\left(t\right)\;p_{3}\left(0\right)=0 \end{array}$$

Note that here the eigenvalues can be in any given order, and we are not assuming that they are ordered in any particular way. We will now investigate *p*_3_. Solving the equation system above reveals that

(9) $$\begin{array}{rcl} p_{3}\left(t\right) & = & \frac{e^{r_{1}t}}{\left(r_{1}-r_{2}\right)\left(r_{1}-r_{3}\right)}+\frac{e^{r_{2}t}}{\left(r_{2}-r_{1}\right)\left(r_{2}-r_{3}\right)}\\ +\;\frac{e^{r_{3}t}}{\left(r_{3}-r_{1}\right)\left(r_{3}-r_{2}\right)} \end{array}$$

Since $e^{r_{3}t}$ is only to be found in *p*_3_(*t*) we have that

(10) $$\begin{array}{r} c_{3}{\text{V}}_{3}e^{r_{3}t}=\frac{1}{\left(r_{3}-r_{1}\right)\left(r_{3}-r_{2}\right)}{\text{M}}_{3}\text{ x}\left(0\right)e^{r_{3}t} \end{array}$$

To get the pre-exponential factor *c*_3_**V**_3_ we write

(11) $$\begin{array}{l} M_{3}x\left(0\right)=\left(A-r_{1}I\right)\left(A-r_{2}I\right)x\left(0\right)\\ \;=\;A\left(Ax\left(0\right)-r_{1}x\left(0\right)-r_{1}x\left(0\right)\right)+r_{1}r_{2}x\left(0\right)\\ \;=\;A\left(\begin{array}{c} -\alpha -r_{1}-r_{2}\\ \alpha \\ 0 \end{array}\right)+\left(\begin{array}{c} r_{1}r_{2}\\ 0\\ 0 \end{array}\right) \end{array}$$

The result above will be a 3 × 1 vector. Since we are solving for *O*(*t*), we are only interested in the second element of this vector. After matrix multiplication, the pre-exponential factor to $e^{r_{3}t}$ in the expression for *O*(*t*) is

(12) $$\begin{array}{r} \frac{\alpha \left(-\alpha -\beta -\gamma -r_{1}-r_{2}\right)}{\left(r_{3}-r_{1}\right)\left(r_{3}-r_{2}\right)}=\frac{\alpha \left(\delta +r_{3}\right)}{\left(r_{3}-r_{1}\right)\left(r_{3}-r_{2}\right)} \end{array}$$

Since the sum of the eigenvalues of a matrix equals the trace, the expression be further reduced as in Equation (12). One of the eigenvalues is always zero so let *r*_1_ = 0, and assume without loss of generality that *r*_3_ > *r*_2_. *r*_3_ is then the slowest decaying term (note that all eigenvalues are non-positive). For some positive value of *t* the following inequalities must hold

(13) $$\begin{array}{r} |c_{3}{\text{V}}_{3}|e^{r_{3}t}>|c_{2}{\text{V}}_{2}|e^{r_{2}t} \end{array}$$

why *c*_3_**V**_3_ will be the dominant term as *t* → ∞. Thus, a peak will exist for *O*(*t*) if the slowest decaying term *r*_3_ has a positive pre-exponential factor, i.e.,

(14) $$\begin{array}{r} \frac{\alpha \left(\delta +r_{3}\right)}{\left(r_{3}-r_{1}\right)\left(r_{3}-r_{2}\right)}>0 \end{array}$$

By the assumption that *r*_1_ > *r*_3_ > *r*_2_ and that α is always positive it follows that this is true exactly when δ + *r*_3_ < 0. Using Vieta's relationships in conjunction with the border condition, *r*_3_ = −δ we can reduce δ + *r*_3_ < 0 to a simpler form. This is due to the fact that there are three equations of the Vieta relationships for det(**A**−*r***I**) = 0 if **A** is a 3 × 3 matrix. In conjunction with *r*_3_ = −δ we have four equations and three eigenvalues, why it is possible to fully eliminate the eigenvalues. Some straight forward algebra reveals that

(15) $$\begin{array}{r} \delta +r_{3}<0\;\Leftrightarrow \;\alpha >\delta \end{array}$$

Thus a sufficient condition for a peak to exists in a three-state scheme is that the opening rate constant α must be greater than the dissociation rate constant δ.

### The existence of a current peak during voltage clamp for a four-state and *n*-state scheme

For the four-state model the solution to *p*_4_(*t*) is obtained by solving the natural extended version of Equation (8), and by the Putzer algorithm we have

(16) $$\begin{array}{r} c_{4}{\text{V}}_{4}e^{r_{4}t}=\frac{1}{\left(r_{4}-r_{1}\right)\left(r_{4}-r_{2}\right)\left(r_{4}-r_{3}\right)}{\text{M}}_{4}\text{x}\left(0\right)e^{r_{4}t} \end{array}$$

To get the pre-exponential factor *c*_4_**V**_4_ we write

(17) $$\begin{array}{l} M_{4}x\left(0\right)=\left(A-r_{1}I\right)\left(A-r_{2}I\right)\left(A-r_{3}I\right)x\left(0\right)\\ \;=A^{2}\left(\begin{array}{c} -2\alpha -r_{1}-r_{2}-r_{3}\\ 2\alpha \\ 0\\ 0 \end{array}\right)+U \end{array}$$

where **U** is some vector with a zero on the third row (due to the tri-diagonal nature of the matrix **A**, the number of non-zero diagonals increase with two for each multiplication and we can omit lower terms than **A**^2^). After matrix multiplication of Equation (17) and taking the third element of the resulting vector we obtain the pre-exponential factor to $e^{r_{4}t}$ in the expression for *O*(*t*),

(18) $$\begin{array}{r} \frac{{2\alpha }^{2}\left(-3\alpha -3\beta -\gamma -r_{1}-r_{2}-r_{3}\right)}{\left(r_{4}-r_{1}\right)\left(r_{4}-r_{2}\right)\left(r_{4}-r_{3}\right)}\\ =\;\frac{2\alpha^{2}\left(\delta +r_{4}\right)}{\left(r_{4}-r_{1}\right)\left(r_{4}-r_{2}\right)\left(r_{4}-r_{3}\right)} \end{array}$$

Again, the expression is reduced using the relationship between the sum of eigenvalues and the trace. Now we can assume without loss of generality that *r*_4_ > *r*_3_ > *r*_2_ and *r*_1_ = 0. As for the three-state model, a peak will exist in the waveform if the pre-exponential factor to the slowest decaying eigenvalue is positive. This is true for a model with an arbitrary number of closed states, since all eigenvalues are real. Then by a sign analysis, the pre-exponential factor to $e^{r_{4}t}$ is positive if, and only if,

(19) $$\begin{array}{r} \delta +r_{4}<0 \end{array}$$

Again using the Vieta's relationships this can be reduced to

(20) $$\begin{array}{r} \delta +r_{4}<0\;\Leftrightarrow \;\alpha >\delta \frac{3+\left(\beta /\alpha \right)+\sqrt{1+6\left(\beta /\alpha \right)+{\left(\beta /\alpha \right)}^{2}}}{4} \end{array}$$

For the interested reader the solution to the general case is given in the Appendix. The technique used in the Appendix is the same as for the three and four state model. It transpires that the pre-exponential factor to $e^{r_{n}t}$ is

(21) $$\begin{array}{r} \frac{\left(n-2\right)!\alpha^{n-2}\left(\delta +r_{n}\right)}{\left(r_{n}-r_{1}\right)\left(r_{n}-r_{2}\right)\ldots \left(r_{n}-r_{n-1}\right)} \end{array}$$

Again, let *r*_*n*_ > *r*_*n*−1_ > ⋯ >*r*_2_ and let *r*_1_ = 0. A similar sign analysis can be performed, yielding that the pre-exponential factor to the slowest decaying eigenvalue is positive exactly when

(22) $$\begin{array}{r} \delta +r_{n}<0 \end{array}$$

**Figure 1:**
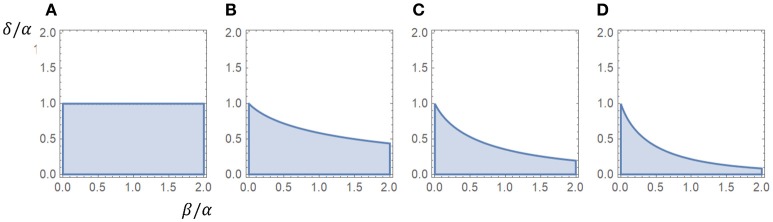
Regions associated with a peak in the β−δ plane (α = 1). The existence of a peak was independent of the value of γ, as long as γ > 0, for all cases. **(A)** One closed state (α > δ). **(B)** Two closed states. **(C)** Three closed states. **(D)** Four closed states.

If β = 0 this criterion is reduced to α > δ. We observe that the influence of β seems to increase with the number of closed states, whereas γ is not involved in the criterion for a peak. The appendix includes a proof that the association rate γ does not affect the criterion for a peak. Figure 1 shows regions associated with the existence of a peak in the β − δ plane, scaled for α.

### Peak height as a function of the rate constants

To analyze the role of the rate constants in the height of the peak, we introduce a factor ψ such that the peak probability *O*_*p*_ can be expressed as

(23) $$\begin{array}{r} O_{p}=\left(1+\psi \right)\;O_{ss} \end{array}$$

where ψ is dependent on the rate constants and *o*_*ss*_ is the steady state open probability in the presence of a blocking agent (see Nilsson et al., 2003). For the three-state model the peak will have its maximum at time

(24) $$\begin{array}{r} t_{p}=\frac{1}{r_{3}-r_{2}}\ln\left(\frac{\delta +r_{2}}{\delta +r_{3}}\right) \end{array}$$

This formula was obtained by taking the time derivate of the solution for *O*(*t*) (see Appendix, A18) and equating this expression with zero. Taking *O*(*t*_*p*_) yields

(25) $$\begin{array}{r} \psi =\frac{\gamma \left(\delta -\alpha \right){\left(\frac{r_{2}+\delta }{r_{3}+\delta }\right)}^{r_{2}/\left(r_{3}-r_{2}\right)}}{\delta \left(r_{3}+\delta \right)} \end{array}$$

Using our analytical solution it is possible to analyze how extreme values of the rate constants affect the peak height. Extreme values are shown in Table 1.

**Table 1:** Limits for ψ for various extremes.

	**i= ∞**	**i= 0**
$\underset{\alpha \to \text{i}}{\text{lim}}\psi (\alpha )$	$\frac{\gamma }{\delta }$	0
$\underset{\beta \to \text{i}}{\text{lim}}\psi (\beta )$	0	$\{\begin{array}{c} \frac{\gamma }{\delta }{\left(\frac{\gamma }{\alpha -\delta }\right)}^{\frac{\gamma +\delta }{\alpha -\gamma -\delta }}\;\alpha \leq \gamma +\delta \\ \frac{\gamma }{\delta }{\left(\frac{\alpha -\delta }{\gamma }\right)}^{-\frac{\gamma +\delta }{\alpha -\gamma -\delta }}\;\alpha \geq \gamma +\delta \end{array}$
$\underset{\gamma \to \text{i}}{\text{lim}}\psi (\gamma )$	$\frac{\alpha }{\delta }-1$	0
$\underset{\delta \to \text{i}}{\text{lim}}\psi (\delta )$	0	∞

In contrast to the criterion for the existence of a peak current, which involves only two rate constants (i.e., α and δ), the peak height depends on all rate constants of Equation (1) (i.e., α, β, γ and δ). To facilitate the use of the derived relationships, we can choose simplifying conditions for the system. Thus, assume that we investigate the blocking effect at high voltage steps (i.e., assuming β = 0 and α > γ + δ) and at a concentration equal to the K_d_-value of the blocking agent (i.e., assuming γ = δ). Algebraically, the following formula is obtained

(26) $$\begin{array}{r} \psi ={\left(\frac{\alpha }{\delta }-1\right)}^{1/\left(1-\alpha /2\delta \right)} \end{array}$$

### Peak height as a function of the voltage

To analyze how the voltage affects the amplitude of the induced peak we used Monte Carlo simulations. Let α and β be described according to Eyring-Polanyi rate theory (Evans and Polanyi, 1935; Eyring, 1935) as

(27) $$\begin{array}{rcl} \alpha & = & ke^{\left(V-V_{1/2}\right)/s} \end{array}$$

(28) $$\begin{array}{rcl} \beta & = & ke^{-\left(V-V_{1/2}\right)/s} \end{array}$$

where *k* is the pre-exponential factor (or the characteristic frequency factor), *V*_1/2_ is the potential for which α = β and *s* is a slope factor describing the influence of the potential. Again, let the concentration of blocking agent be at K_d_-value (i.e., γ = δ).

**Figure 2:**
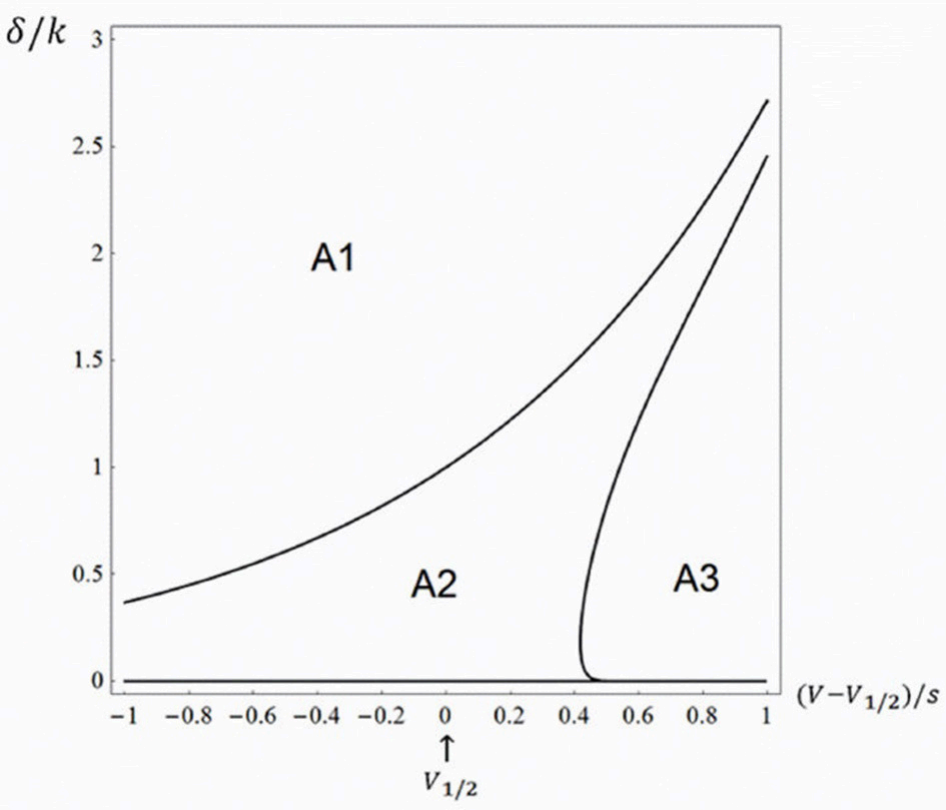
Topologically equivalent regions in the rate-potential plane. The three-state model (Equation 1) at K_d_-concentration of the blocking drug (i.e., γ = δ). A1 represents an area in which there is no open probability peak and the steady state value decreases with potential approaching a value of a half, A2 represents an area in which there is a peak and the relative amplitude decreases with potential and A3 represents an area in which there is a peak, but the relative amplitude increases with potential, approaching a value of one.

Using Monte Carlo simulations in conjunction with analytical tools, we found three different regions in the δ−*V* plane: (i) One area, which we call A1, in which there is no open probability peak; (ii) a second area, A2, in which there is a peak and the relative amplitude (peak open probability relative to steady state with no blocking agent) decreases with potential and (iii) a third area, A3, in which there is a peak, but the relative amplitude increases with potential, approaching a value of one. Area A1 was defined using the criteria obtained above, i.e. α > δ. The border between A2 and A3 was obtained in the following way. Using custom written software in Mathematica 11.0 (Wolfram Inc.), we evenly assigned values using the built-in function *RandomReal* in parameter space of the right half of Figure 2 and investigated the voltage dependence (see Equations 26–28). The border between A2 and A3 were drawn using the built-in function *ContourPlot*. Figure 2 shows the three topological regions.

The findings in Figure 2 are to some extent congruent with the findings of the relatively few experimental investigations of local anesthetic effects on non-inactivating Kv channels (see e.g. the effect of bupivacaine on Kv1.5 channels, described by Gonzalez et al. (2001), where the relative peak amplitude goes from a potential region where there is no peak, i.e., corresponding to A1, and to a potential region where the relative peak increases with potential, i.e., corresponding to A3). Clearly this issue requires further experimental investigations to be clarified.

## Discussion

The present analysis was undertaken to derive general principles of kinetics from the models of binding mechanisms. In previous studies, we showed that the action of the local anesthetic bupivacaine on a non-inactivating potassium channel could be described by Markov chain models, assuming that binding occurs exclusively to channels in open state (Nilsson et al., 2003, 2008).

In the present study, we mathematically analyzed Markov models, with special reference to the question of the existence of a peak and its voltage dependence. We (i) derived the criterion for the existence of a peak in the open probability time evolution for an open-state binding kinetic scheme, comprising one and two closed states (Schemes 1 and 2.1), (ii) derived the criterion for a peak in an open-state kinetic scheme with an arbitrary number of closed states, (iii) derived formula for the relative height and the block of the peak amplitude for Scheme 1, and (iv) determined (by Monte Carlo simulations) the voltage dependence of the relative peak block for Scheme 1.

Contemplating these findings, we note the important role that the dissociation rate constant plays for the open probability peak features. Intuitively, one would have expected that the on rate (γ) would be the key player in the existence of a peak. Nevertheless, we find that the association (binding) rate constant is the only rate constant that does *not* influence the existence of a peak. In a three-state model (Scheme 1), the existence of a peak is given by the simple relation α > δ (i.e., the activation rate constant is greater than the dissociation (unbinding) rate constant). If β = 0 (the deactivation rate constant), this relationship holds for models with a higher number of closed states. Generally, a peak exists if −*r*_*n*_ > δ where *r*_*n*_ is the eigenvalues closest to zero (i.e. the slowest decaying term).

Additionally, we could determine a topological region in the dissociation rate–voltage plane for Scheme 1 that is characterized by a decreasing block of the induced peak with potential.

Using analytical and Markov chain models that describe the action of blocking drugs on ion channels, we could derive general principles of linear three- and higher-state schemes. Thus, we could show that the existence of a current peak for open state binding schemes mainly depends on the dissociation (unbinding) rate constant δ, the criterion for a scheme with one closed state (Scheme 1) being α > δ (i.e., the activation rate constant should be greater than the dissociation (unbinding) rate constant). We could also show that different peak amplitude-voltage relations characterize specific topological regions of the dissociation rate–voltage plane for Scheme 1, a finding that still awaits experimental corroboration.

Understanding the relationships between the rates and the dynamics of the block of ion channels is essential for understanding how more drugs modulate neuronal firing patterns and thus how they function in pain, epilepsy, arrhythmia and anesthesia; these insights are crucial when developing anti-epileptic, anti-arrhythmic and anesthetic drugs. Furthermore, it should be noted that many pharmacological processes other than drug binding to ion channels are analysable in terms of serial Markov chains, and thus, these pharmacological processes are constrained by the mathematical expression derived from the present study.

## Author contributions

HZ, JN, and PÅ: designed the research; HZ and PÅ: performed the mathematical work; HZ, JN, and PÅ: analyzed the results; and HZ, JN, and PÅ: wrote the manuscript.

### Conflict of interest statement

The authors declare that the research was conducted in the absence of any commercial or financial relationships that could be construed as a potential conflict of interest. The reviewer AS and handling Editor declared their shared affiliation.

## References

[B1] Colquhoun D. Hawkes A. (1995). The principles of the stochastic interpretation of ion-channel mechanisms, in Single-Channel Recording, eds Sakmann B. Neher E. (New York, NY: Springer US), 397–482.

[B2] Evans M. G. Polanyi M. (1935). Some applications of the transition state method to the calculation of reaction velocities, especially in solution. Trans. Faraday Soc. 31, 875–894. 10.1039/tf9353100875

[B3] Eyring H. (1935). The activated complex and the absolute rate of chemical reactions. Chem. Rev. 17, 65–77. 10.1021/cr60056a006

[B4] Fitzhugh R. (1961). Impulses and physiological states in theoretical models of nerve membrane. Biophys. J. 1, 445–466. 10.1016/S0006-3495(61)86902-6 19431309 PMC1366333

[B5] Gonzalez T. Longobardo M. Caballero R. Delpon E. Tamargo J. Valenzuela C. (2001). Effects of bupivacaine and a novel local anesthetic, IQB-9302, on human cardiac K^+^ channels. J. Pharmacol. Exp. Ther. 296, 573–583. 11160646

[B6] Gouwens N. W. Zeberg H. Tsumoto K. Tateno T. Aihara K. Robinson H. P. (2010). Synchronization of firing in cortical fast-spiking interneurons at gamma frequencies: a phase-resetting analysis. PLoS Comput. Biol. 6:e1000951. 10.1371/journal.pcbi.1000951 20941393 PMC2947988

[B7] Izhikevich E. M. (2007). Dynamical Systems in Neuroscience. Cambridge, MA: MIT Press.

[B8] Johnston D. Wu S. M. S. (1995). Foundations of Cellular Neurophysiology. Cambridge, MA: MIT Press Cambridge.

[B9] Koch C. (2004). Biophysics of Computation: Information Processing in Single Neurons. New York, NY: Oxford University Press.

[B10] Longobardo M. Gonzalez T. Caballero R. Delpon E. Tamargo J. Valenzuela C. (2001). Bupivacaine effects on hKv1.5 channels are dependent on extracellular pH. Br. J. Pharmacol. 134, 359–369. 10.1038/sj.bjp.0704251 11564654 PMC1572951

[B11] Nilsson J. Madeja M. Arhem P. (2003). Local anesthetic block of Kv channels: role of the S6 helix and the S5-S6 linker for bupivacaine action. Mol. Pharmacol. 63, 1417–1429. 10.1124/mol.63.6.1417 12761353

[B12] Nilsson J. Madeja M. Elinder F. Arhem P. (2008). Bupivacaine blocks N-type inactivating Kv channels in the open state: no allosteric effect on inactivation kinetics. Biophys. J. 95, 5138–5152. 10.1529/biophysj.108.130518 18790854 PMC2586568

[B13] Putzer E. J. (1966). Avoiding the jordan canonical form in the discussion of linear systems with constant coefficients. Am. Math. Monthly 73, 2–7. 10.2307/2313914

[B14] Sahlholm K. Zeberg H. Nilsson J. Ogren S. O. Fuxe K. Arhem P. (2016). The fast-off hypothesis revisited: A functional kinetic study of antipsychotic antagonism of the dopamine D2 receptor. Eur. Neuropsychopharmacol. 26, 467–476. 10.1016/j.euroneuro.2016.01.001 26811292

[B15] Zeberg H. Blomberg C. Århem P. (2010). Ion channel density regulates switches between regular and fast spiking in soma but not in Axons. PLoS Comput. Biol. 6:e1000753 10.1371/journal.pcbi.1000753 20421932 PMC2858683

[B16] Zeberg H. Robinson H. P. Århem P. (2015). Density of voltage-gated potassium channels is a bifurcation parameter in pyramidal neurons. J. Neurophysiol. 113, 537–549. 10.1152/jn.00907.2013 25339708 PMC4297787

