# Supplementary material for: The Importance of the Dissociation Rate in Ion Channel Blocking
Source: Front Cell Neurosci. 2018 Feb 9;12:33. doi: 10.3389/fncel.2018.00033 (PMC5811512; doi:10.3389/fncel.2018.00033)
Supplement: Supplementary file 1 [file Appendix.pdf]

APPENDIX

The Importance of the Dissociation Rate in Ion Channel Blocking

Front. Cell. Neurosci. 12:33. doi: 10.3389/fncel.2018.00033

Hugo Zeberg[1,*], Johanna Nilsson[1,2] and Peter Århem[1]
Department of Neuroscience, Karolinska Institutet, 171 77 Stockholm, Sweden
Department of Clinial Neuroscience, Karolinska Institutet, 171 76 Stockholm, Sweden
**hugo.zeberg@ki.se*

Condition for a Peak in the General Case with a $n \times n$ Matrix

By the Putzer algorithm, the solution to $\mathbf{x}' = \mathbf{Ax}$ can be written as

$$\mathbf{x}(t) = (p_1(t)\mathbf{M}_1 + p_2(t)\mathbf{M}_2 + \cdots + p_n(t)\mathbf{M}_n)\mathbf{x}_0 \tag{A1}$$

where p_i and $\mathbf{M}_i$ are defined as follows. Define $n \times n$ matrices $\mathbf{M}_1, \ldots, \mathbf{M}_n$ by the formulae

$$\mathbf{M}_1 = I \qquad \mathbf{M}_i = (\mathbf{A} - r_{i-1}I)\mathbf{M}_{i-1} \quad i = 2, \ldots, n \tag{A2}$$

and let the functions $p_1, \ldots, p_n$ be given by solutions to the differential system

$$\begin{aligned} p_1{}' &= r_i p_1 & p_1(0) &= 1 \\ p_2{}' &= r_2 p_2 - p_1 & p_2(0) &= 0 \\ &\vdots \\ p_n{}' &= r_n p_n - p_{n-1} & p_n(0) &= 0 \end{aligned} \tag{A3}$$

where r_i are eigenvalues in any arbitrary order to the matrix $\mathbf{A}$. We will begin by proving that

$$p_i(t) = \sum_{j=1}^{j=i} \left(\prod_{\substack{k=1 \\ k \neq j}}^{k=i} (r_j - r_k) \right)^{-1} e^{r_j t}. \tag{A4}$$

It is straight forward to varify that this is a solution to the equation system above.

$$\begin{aligned} p_i'(t) - r_i p_i(t) &= \sum_{j=1}^{j=i} r_j \left(\prod_{\substack{k=1 \\ k \neq j}}^{k=i} (r_j - r_k) \right)^{-1} e^{r_j t} - \sum_{j=1}^{j=i} r_i \left(\prod_{\substack{k=1 \\ k \neq j}}^{k=i} (r_j - r_k) \right)^{-1} e^{r_j t} \\ &= \sum_{j=1}^{j=i} (r_j - r_i) \left(\prod_{\substack{k=1 \\ k \neq j}}^{k=i} (r_j - r_k) \right)^{-1} e^{r_j t} \\ &= \sum_{j=1}^{j=i-1} \left(\prod_{\substack{k=1 \\ k \neq j}}^{k=i-1} (r_j - r_k) \right)^{-1} e^{r_j t} \\ &= p_{i-1} \end{aligned} \tag{A5}$$

It is also necessary to verify that $p_i(0) = 0$ for $i > 1$. We will show this using induction. By change of variable, $s_j = r_j - r_i$, we can show that if $p_{i-1}(0) = 0$ then so is $p_i(0)$

$$
\begin{aligned}
p_i'(0) - r_i p_i(0) &= 0 \\
\sum_{j=1}^{j=i} r_j \left(\prod_{\substack{k=1 \\ k \neq j}}^{k=i} (r_j - r_k) \right)^{-1} - r_i p_i(0) &= 0 \\
\sum_{j=1}^{j=i} (s_j + r_i) \left(\prod_{\substack{k=1 \\ k \neq j}}^{k=i} (s_j - s_k) \right)^{-1} - r_i p_i(0) &= 0 \\
\sum_{j=1}^{j=i} (s_j + r_i) \left(s_j \prod_{\substack{k=1 \\ k \neq j}}^{k=i-1} (s_j - s_k) \right)^{-1} - r_i p_i(0) &= 0 \\
(1 + r_i) p_{i-1}(0) - r_i p_i(0) &= 0 \\
p_i(0) &= 0,
\end{aligned}
\tag{A6}
$$

completing the proof of the analytic expression for $p_i(t)$. Note that for $i = 2$ the third step is not permissible, but $p_2(0)$ is trivially zero and can serve as the basis for the induction. We now turn to the matrix differential equation of interest. An *n*-state scheme with *n-2* closed states written in matrix form $\mathbf{x}' = \mathbf{A}\mathbf{x}$ where

$$
\mathbf{x} = \begin{bmatrix} C_1(t) \\ C_2(t) \\ \vdots \\ C_{n-2}(t) \\ O(t) \\ B(t) \end{bmatrix}
\quad
\mathbf{A} = \begin{bmatrix}
-(n-2)\alpha & \beta & 0 & 0 & \cdots & 0 \\
(n-2)\alpha & -(n-3)\alpha - \beta & 2\beta & \ddots & \ddots & \vdots \\
0 & (n-3)\alpha & \ddots & \ddots & \ddots & 0 \\
0 & \ddots & \ddots & \ddots & (n-2)\beta & 0 \\
\vdots & \ddots & \ddots & \alpha & -(n-2)\beta - \gamma & \delta \\
0 & \cdots & 0 & 0 & \gamma & -\delta
\end{bmatrix}
\tag{A7}
$$

with the general solution

$$
\mathbf{x}(t) = c_1 \mathbf{V}_1 e^{r_1 t} + c_2 \mathbf{V}_2 e^{r_1 t} \dots + c_n \mathbf{V}_n e^{r_n t} \tag{A8}
$$

where c_i are constant depending on the initial condition and $\mathbf{V}_i$ are the corresponding eigenvectors to $\mathbf{A}$. Let $\mathbf{x}(0) = [1 \quad 0 \quad \cdots]^{\mathbf{T}}$ (i.e. the channels have an initial probability of one in beeing in the first closed state). Using Putzers algorithm and the result for $p_i(t)$ we can write

$$
c_n \mathbf{V}_n e^{r_n t} = \left(\prod_{k=1}^{k=n-1} (r_n - r_k) \right)^{-1} \mathbf{M}_n \mathbf{x}(0) e^{r_n t} \tag{A9}
$$

Note that r_n only exists in $p_n(t)$ and that we can extract the last term in the sum giving the analytical expression for $p_n(t)$.

To get the pre-exponential factor $c_n\mathbf{V}_n$ we need to solve $\mathbf{M}_n\mathbf{x}(0)$,

$$\begin{aligned}\mathbf{M}_n\mathbf{x}(0) &= (\mathbf{A}-r_1\mathbf{I})(\mathbf{A}-r_2\mathbf{I})\ldots(\mathbf{A}-r_{n-1}\mathbf{I})\mathbf{x}(0)\\ &= \mathbf{A}^{n-2}(\mathbf{A}-r_1\mathbf{I}-r_2\mathbf{I}\ldots-r_{n-1}\mathbf{I})\mathbf{x}(0)+\mathbf{U}\\ &= \mathbf{A}^{n-2}\begin{pmatrix} a_{1,1}-r_1-r_2-\cdots-r_{n-1}\\ a_{2,1}\\ 0\\ \vdots\\ 0\end{pmatrix}+\mathbf{U}\end{aligned} \tag{A10}$$

where $a_{i,j}$ are elements of the matrix $\mathbf{A}$, $\mathbf{I}$ the identity matrix, and $\mathbf{U}$ is some vector with a zero on the next last row (due to the tridiagonal nature of the matrix $\mathbf{A}$, the number of non-zero diagnoals increase with two for each multiplication why we can omit terms involving lower powers than $n-2$). Given that we are solving for $O(t)$ we are only interested in the next last element of the resulting vector. Moreover, due to the zeros of the column vector containing the eigenvalues it suffices to obtain $(\mathbf{A}^{n-2})_{n-1,1}$ and $(\mathbf{A}^{n-2})_{n-1,2}$. We begin with the leftmost element, viz.

$$\begin{aligned}(\mathbf{A}^{n-2})_{n-1,1} &= (\mathbf{A})_{n-1,:}(\mathbf{A}^{n-3})_{:,1}\\ &= (\mathbf{A})_{n-1,:}\big(a_{2,1}(\mathbf{A}^{n-4})_{:,2}\big) = (\mathbf{A})_{n-1,:}\big(a_{2,1}a_{3,2}(\mathbf{A}^{n-5})_{:,3}\big) = \cdots\\ &= \prod_{i=1}^{i=n-2} a_{i+1,i}\end{aligned} \tag{A11}$$

Again, we can omit terms due to the sparse nature of $\mathbf{A}$. We are now left with obtaining an analytic expression for $(\mathbf{A}^{n-2})_{n-1,2}$.

$$\begin{aligned}(\mathbf{A}^{n-2})_{n-1,2} &= (\mathbf{A})_{n-1,:}(\mathbf{A}^{n-3})_{:,2}\\ &= (\mathbf{A})_{n-1,:}\big(a_{2,2}(\mathbf{A}^{n-4})_{:,2}+a_{3,2}(\mathbf{A}^{n-4})_{:,3}\big)\\ &= (\mathbf{A})_{n-1,:}\Big(a_{2,2}a_{3,2}(\mathbf{A}^{n-5})_{:,3}+a_{3,2}\big(a_{3,3}(\mathbf{A}^{n-5})_{:,3}+a_{4,3}(\mathbf{A}^{n-5})_{:,4}\big)\Big)\\ &= \left(\prod_{i=2}^{i=n-2} a_{i+1,i}\right)\left(\sum_{i=2}^{i=n-1} k_{i,i}\right)\end{aligned} \tag{A12}$$

Now using the fact that the sum of eigenvalues equals the trace we multiply $(\mathbf{A}^{n-2})_{n-1,1}$ and $(\mathbf{A}^{n-2})_{n-1,2}$ with the respective value in the column vector in eqn. A10 yielding

$$\begin{aligned}&\left(\prod_{i=1}^{i=n-2} a_{i+1,i}\right)\big(a_{1,1}-\mathrm{Tr}(\mathbf{A})+r_n\big)+\left(\prod_{i=2}^{i=n-2} a_{i+1,i}\right)\big(\mathrm{Tr}(\mathbf{A})-a_{1,1}-a_{n,n}\big)a_{2,1}\\ &= \left(\prod_{i=1}^{i=n-2} a_{i+1,i}\right)\big(r_n-a_{n,n}\big)\\ &= (n-2)!\,\alpha^{n-2}(\delta+r_n)\end{aligned} \tag{A13}$$

Combining with our result for $p_n(t)$ and we obtain

$$(n-2)!\,\alpha^{n-2}(\delta+r_n)\left(\prod_{k=1}^{k=n-1}(r_n-r_k)\right)^{-1} e^{r_n t} \tag{A14}$$

Assume without loss of generality that r_n is the slowest decaying term. (r_n-r_k) will be strictly positive except for the eigenvalue which is zero. Thus,

$$\left(\prod_{k=1}^{k=n-1}(r_n-r_k)\right)^{-1} < 0 \tag{A15}$$

By the fact that the rate constants are positive we have $(n-2)!\,\alpha^{n-2} > 0$. Hence, the pre-exponetial factor is positive if, and only if,

$$\delta + r_n < 0. \tag{A16}$$

By elimination of the eigenvalues from Vieta's formulae it follows that $(r_n+\delta) < 0$ is a root to

$$\sum_{i=1}^{i=n}(-1)^n \frac{b_i}{b_n}\delta^i < 0 \tag{A17}$$

where b_i are the coefficitens to the charactersitic polynomial to $\mathbf{A}$. By the Picard–Lindelöf theorem, the solution to $\mathbf{x}' = \mathbf{A}\mathbf{x}$ is unique. Thus, since we are free to chose the eigenvalues in any given order, our result for the prexpoenential factor to $e^{r_n t}$ must be true for any $e^{r_i t}$ why $O(t)$ can be written on a surprisingly compact form, namely

$$O(t) = \sum_{i=1}^{i=n}\frac{(n-2)!\,\alpha^{n-2}(r_i+\delta)}{\prod_{i\neq j}(r_i-r_j)}e^{r_i t} \tag{A18}$$

It transpires that the associated rate γ does the not affect the prerequisites for a peak. Let $\delta = -r_n$, which gives

$$\begin{aligned}
&\det(A - r_n I) = \det(A+\delta I) \\
&= \det\begin{pmatrix}
-(n-2)\alpha+\delta & \beta & 0 & 0 & \cdots & 0 \\
(n-2)\alpha & -(n-3)\alpha-\beta+\delta & 2\beta & \ddots & \ddots & \vdots \\
0 & (n-3)\alpha & \ddots & \ddots & \ddots & 0 \\
0 & \ddots & \ddots & \ddots & (n-2)\beta & 0 \\
\vdots & \ddots & \ddots & \alpha & -(n-2)\beta-\gamma+\delta & \delta \\
0 & \cdots & 0 & 0 & \gamma & 0
\end{pmatrix} \\
&= 0
\end{aligned} \tag{A19}$$

The determinant can now be expanded in terms of minors along the last row, yielding

$$\gamma \det \begin{pmatrix} -(n-2)\alpha+\delta & \beta & 0 & 0 & 0 \\ (n-2)\alpha & -(n-3)\alpha-\beta+\delta & 2\beta & \ddots & \vdots \\ 0 & (n-3)\alpha & \ddots & \ddots & 0 \\ 0 & \ddots & \ddots & \ddots & 0 \\ \vdots & \ddots & \ddots & \alpha & \delta \end{pmatrix} = 0 \tag{A20}$$

γ can in this equation be factored out, showing that γ does not influence the existance of a peak as long as $\gamma > 0$.
